# Enlargements of the Liver.—VI

**Published:** 1910-02-05

**Authors:** 


### Medicine.

### ENLARGEMENTS OF THE LIVER.—VI.

CIRRHOSIS (*concluded*).

### Syphilitic Cirrhosis.

(*a*) *Intralobular, intercellular, or pericellular.*—This form is most frequently a manifestation of congenital syphilis. The liver is enlarged and hard, but of normal shape. The capsule may or may not be adherent to the diaphragm. In colour the organ may be normal, or pale and yellowish, or reddish-grey. On section it is tough and bloodless. It is not always easy to say with the naked eye whether there is any fibrosis or not, but sometimes the cut surface presents a peculiar finely streaked, marbled or mottled appearance, a close inspection showing greyish streaks and small pale spots resembling minute miliary tubercles. Microscopically a fibrosis and considerable small celled infiltration may be seen between the individual cells, many of which show cloudy swelling, fat globules, or other degenerative changes.

During life a physical examination may determine that the liver is uniformly enlarged with a hard, smooth, and possibly tender surface and a sharp, well-defined edge. The spleen may also be found to be increased in size. The symptoms are very variable. Jaundice and ascites are rare. Children thus affected generally die from marasmus. The diagnosis rests on the association of uniform enlargement of the liver with other signs of congenital syphilis.

### Gummatous Cirrhosis.

This form of cirrhosis usually occurs as a late tertiary manifestation of acquired syphilis. By some the term cirrhosis is denied to it, gummatous fibrosis being used for it instead. The disease begins with the formation of multiple gummata throughout the liver, followed by cicatrisation, so that the whole organ, or the greater part of it, may become intersected with bands of fibrous tissue. In consequence of the irregular distribution and contraction of the latter the organ becomes deformed, the surface being irregular, scarred, and lobular, like the outline of a bunch of grapes.

On making a physical examination during life the liver is not always to be felt, but occasionally it is enlarged and its surface is extremely irregular and nodular. It is apt to be mistaken for secondary carcinoma of the liver. If the fibrous tissue has invaded the portal vein or its main branches there may be all the signs of portal obstruction—ascites hæmatemesis, and so forth; and if the bile ducts are, constricted, jaundice. In all cases of cirrhosis with ascites and enlargement of the liver the possibility of syphilis should be considered and carefully investigated on account of its important bearing on the treatment. Lack of improvement when mercurials and potassium iodide are given does not prove that the lesion is not syphilitic; but when syphilis is the cause, then it sometimes happens that iodides and mercury alleviate the symptoms. It depends upon the extent to which the gummata have already become fibrosed before the treatment is begun.

### Leuchæmia.

The liver may be much enlarged in either form of leuchæmia. It may weigh as much as 13 lbs. (Welch). The surface is smooth and the edge sharp and well-defined. On section the hepatic tissue generally looks paler than normal, and in some cases a close inspection may show the presence of a number of small pale soft areas resembling miliary tubercles. Microscopically a general diffuse infiltration with leucocytes is to be seen, the columns of liver cells being separated from each other by layers of leucocytes.

The enlargement of the liver in the spleno-medullary form is generally over-shadowed by the enormous increase in the size of the spleen, which may reach down to the pelvis.

The chief signs of the disease are anæmia, hæmorrhages from the mucous membranes, and progressive enlargement of the spleen. An accurate diagnosis can only be made after a careful and systematic examination of the blood which shows enormous leucocytosis reaching from 100,000 white cells per cubic millimetre of blood to even 1,000,000. A differential leucocyte count will show the presence of a large number of myelocytes (20 to 60 per cent.) in the splenomedullary form, and upwards of 90 per cent. of lymphocytes in the lymphatic form. Treatment is mainly by means of arsenic and the *x*-rays.

### Hodgkin's Disease, or Lymphadenoma.

In Hodgkin's disease, or lymphadenoma, the liver is not infrequently slightly or moderately enlarged. The surface is usually smooth, but in exceptional cases it is nodular, when there are large lymphadenomatous deposits present in the organ. The edge is sharp and well-defined. On section the organ is paler than normal, and irregularly shaped masses of lymphadenomatous structure may be seen scattered throughout its substance. A microscopical examination shows the presence of large collections of lymphoid cells between the lobules of the liver.

The chief clinical signs of the disease are: Anæmia, enlarged glands, especially in the neck and axillæ, the skin over them being unaffected, moderate or considerable enlargement of the spleen, slight enlargement of the liver, periods of pyrexia alternating with periods during which the patient's temperature is normal, or nearly so, and in the later stages a tendency to various hæmorrhages and to effusions into the serous cavities. An examination of the blood shows nothing distinctive; there will be a diminution of the red blood corpuscles and a decrease in the hæmoglobin, the latter being more diminished than are the red cells, so that the colour index is less than 1; the leucocytes are usually normal in numbers or slightly diminished, and the differential leucocyte count may either lie within the normal limits or else exhibit a variable degree of relative lymphocytosis.

(*To be continued.*)

